# Cytotoxic Lesions of the Corpus Callosum (CLOCC) in Siblings: A Case Report

**DOI:** 10.2174/0115734056410886250924052841

**Published:** 2025-10-03

**Authors:** Qihong Chen, Jinqi Huang, Jianfang Huang

**Affiliations:** 1 Department of Interventional Vascular Surgery, The First Hospital of Putian City, No. 449 Nanmenxi Road, Chengxiang District, Putian, 351100, Fujian Province, People's Republic of China; 2 Department of Medical Imaging, The First Hospital of Putian City, No. 449 Nanmenxi Road, Chengxiang District, Putian, 351100, Fujian Province, People's Republic of China

**Keywords:** Siblings, Corpus callosum, Encephalitis, Brain diseases, Case reports

## Abstract

**Introduction/Background:**

Cytotoxic lesions of the corpus callosum (CLOCC) are a rare clinical-radiological syndrome with an unclear specific pathogenesis, and cases occurring consecutively in siblings are exceptionally uncommon. This study reports two pediatric siblings with CLOCC (one experiencing two episodes), highlighting the potential role of genetic susceptibility in its pathogenesis. The findings contribute to the limited literature on familial CLOCC and recurrent cases in children.

**Case Presentation:**

Two brothers (aged 9 and 12) presented with sudden-onset aphasia and unilateral limb weakness, preceded by rhinorrhea. Magnetic resonance imaging (MRI) revealed reversible lesions in the splenium of the corpus callosum and bilateral frontoparietal white matter, consistent with CLOCC. Both patients received immunomodulatory therapy (*e.g.*, corticosteroids, intravenous immunoglobulin) and symptomatic treatment, achieving full neurological recovery within approximately one week. The elder sibling had a recurrence two years later (when the patient was 14 years old) with similar imaging findings. Laboratory tests ruled out common infections, and cerebrospinal fluid analysis was unremarkable.

**Conclusion:**

This case underscores CLOCC as a heterogeneous condition with possible genetic predisposition, as evidenced by its occurrence in siblings and recurrence in one sibling. While prognosis is generally favorable, the observed sibling clustering and individual recurrence suggest the need for further research into underlying genetic or immunological mechanisms.

## 1 INTRODUCTION

In our practice, we observed two pediatric siblings successively afflicted with cytotoxic lesions of the corpus callosum (CLOCC), with one of the patients experiencing two episodes. CLOCC was previously described under various terms, such as mild encephalitis/encephalopathy with a reversible splenial lesion (MERS), reversible splenial lesion syndrome (RESLES), or transient lesions of the splenium of the corpus callosum. In this article, we collectively adopt the term CLOCC to more accurately reflect the underlying pathophysiological mechanisms and to emphasize that these lesions are not limited exclusively to the splenium of the corpus callosum. To our knowledge, cases of CLOCC in siblings are extremely rare worldwide, with only a few individual reports. For instance, Imamura, [1] Tahara, [2] Gatto *et al.* [3] each reported cases of CLOCC affecting siblings, either sisters or brothers. Research on recurrent CLOCC is also extremely limited, with only sporadic case reports currently available. For example, Xue *et al.* described two children with recurrent CLOCC, each experiencing a different number of relapses [4]. Given the rarity and research value of these cases, we hereby present this case report, aiming to contribute more empirical evidence to the study of CLOCC.

## 2 CASE PRESENTATION

### 2.1 Case 1

**Fig. (1):**
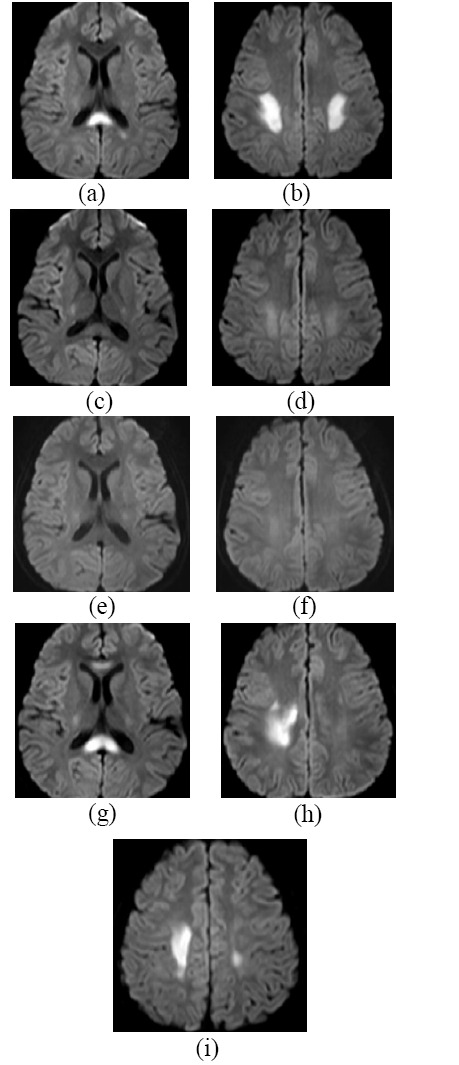
Male patient with cytotoxic lesions of the corpus callosum. **a, b.** Diffusion weighted imaging (DWI) on March 26^th^, 2014, showing abnormal high signal in the splenium of the corpus callosum and the deep white matter at the junction of the bilateral fronto-parietal lobes; **c, d.** On April 5^th^, 2014, the abnormal high signal on DWI was significantly reduced; **e, f.** Follow-up DWI on June 27^th^, 2015, appeared normal; **g**-**i**. DWI on March 13^th^, 2016, revealed an abnormally high signal in the splenium and genu of the corpus callosum, as well as the deep white matter at the junction of the bilateral fronto-parietal lobes (more prominent on the right side).

A 12-year-old male was admitted to the hospital on March 26^th^, 2014, due to “sudden aphasia accompanied by left-sided limb weakness for half a day”. He had no significant medical history, but reported a history of “runny nose” one week prior and a “fall on the stairs” five days prior, for which he did not seek medical attention. Upon physical examination, the patient was unable to speak. Muscle strength was Grade 3 in the left limbs and Grade 4 in the right limbs. Muscle tone was normal. The left knee jerk and Achilles tendon reflex were slightly diminished, and the left Babinski sign was positive. The white blood cell count was 3.48×10^9^/L (normal range: 3.5-9.5×10^9^/L), the platelet count was 76×10^9^/L (normal range:125-350×10^9^/L), and the C-reactive protein (CRP) level was 1.06 mg/L (normal range: 0-8 mg/L). Blood chemistry was generally normal, with a serum sodium level of 139.0 mmol/L (normal range: 137-147 mmol/L). Blood tests for M antibodies (against *Toxoplasma gondii*, rubella virus, cytomegalovirus, and herpes simplex virus type II) were negative. Fecal bacterial culture was also negative. Cranial magnetic resonance imaging (MRI) revealed abnormal signal in the splenium of the corpus callosum and the deep white matter at the junction of the bilateral fronto-parietal lobes. Diffusion weighted imaging (DWI) showed high signal, T1 weighted imaging (T1WI) demonstrated low signal, T2 weighted imaging (T2WI) exhibited high signal, and fluid-attenuated inversion recovery (FLAIR) sequences also showed high signal (Fig. **1a**, **b**). In terms of treatment, the patient was administered mannitol, human immunoglobulin, methylprednisolone sodium succinate, omeprazole, and other therapies. Electroencephalogram (EEG) on March 27^th^ showed abnormal EEG for a child, with diffuse theta wave activity observed across multiple leads; Electromyography showed peripheral neuropathic damage in both upper and lower limbs. On March 28^th^, a lumbar puncture was performed, and the cerebrospinal fluid (CSF) routine test (white blood cell count was 4.00×10^6^/L, with a normal range of 0 - 8×10^6^/L), biochemistry, M antibodies (against *Toxoplasma gondii*, rubella virus, cytomegalovirus, and herpes simplex virus type II), bacterial smear, and bacterial culture showed no significant abnormalities. On March 30^th^, the patient experienced dizziness and vomiting and was given symptomatic treatment. A follow-up cranial MRI on April 5^th^ showed a significant reduction in abnormal signals (Fig. **1c**, **d**). The patient was discharged on April 7^th^ after making a full recovery. A subsequent cranial MRI on June 27^th^, 2015, revealed no abnormalities (Fig. **1e**, **f**).

On March 11^th^, 2016, when the patient was 14 years old, he was readmitted to the hospital with a complaint of “left-sided limb weakness for one day.” The muscle strength of the left limbs was Grade 4. The blood routine and biochemistry tests were generally normal, with a serum sodium level of 141.0 mmol/L. During the hospitalization, the patient gradually developed a cough, and the bacterial culture of the nasopharyngeal swab was negative. On March 12^th^, the chest X-ray showed no significant abnormalities. On March 13^th^, cranial MRI revealed abnormal signal in most of the corpus callosum and the white matter of the bilateral corona radiata, which exhibited high signal on DWI, low on T1WI, high on T2WI, and high on FLAIR (Fig. **1g**-**i**). The patient was treated with human immunoglobulin, prednisone, cerebroprotein hydrolysate, and pholcodine, among other therapies. On March 17^th^, the patient's symptoms alleviated, and he was discharged from the hospital.

### 2.2 Case 2 (Younger Brother of Case 1)

**Fig. (2):**
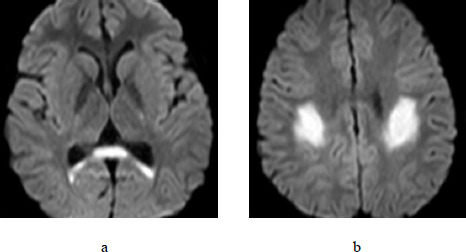
Male, 9 years old, with cytotoxic lesions of the corpus callosum. **a, b.** On June 18^th^, 2015, Diffusion Weighted Imaging showed an abnormally high signal in the splenium of the corpus callosum and the deep white matter at the junction of the bilateral fronto-parietal lobes.

A 9-year-old male was admitted on June 17^th^, 2015, with sudden aphasia and right-sided limb weakness for 2 hours. He had no significant past medical history but reported symptoms of nasal congestion and rhinorrhea one week prior. Physical examination revealed inability to speak, muscle strength of Grade 4 in the left limbs and Grade 3 in the right limbs, normal muscle tone, and slightly decreased bilateral knee and Achilles tendon reflexes. The white blood cell count was 8.94×10^9^/L, CRP was 0.5 mg/L, and blood chemistry was generally normal. Blood sodium level was 144.0 mmol/L. Serological tests for M antibodies (against *Toxoplasma gondii*, rubella virus, cytomegalovirus, and herpes simplex virus type II) were negative, as were tests for adenovirus and coxsackievirus antibodies. Fecal bacterial culture was negative. Cranial computed tomography scan demonstrated a low-density lesion in the right semi-oval center. For treatment, the patient was administered mannitol, methylprednisolone sodium succinate, human immunoglobulin, omeprazole, and other therapies. On June 18^th^, cranial MRI revealed abnormal signal in the splenium of the corpus callosum, the posterior part of the body of the corpus callosum, as well as in the deep white matter at the junction of the bilateral fronto-parietal lobes, characterized by high signal intensity on DWI, low on T1WI, high on T2WI, and high on FLAIR (Fig. **2**). All CSF tests were essentially normal (White blood cell count was 4.00×10^6^/L). On June 21^st^, the patient's speech was clear and able to form sentences, with muscle strength of Grade 4 in both limbs. EEG showed abnormal dynamic EEG for a child, with diffuse theta wave activity observed across various electrodes during the waking state. On June 24^th^, the patient developed fever and vomiting, with a maximum body temperature of 39.4°C, and was given symptomatic treatment. On June 25^th^, the patient's speech was clear, muscle strength in both limbs was normal, white blood cell count was 10.67×10^9^/L, and CRP was 9.58 mg/L. Treatment with piperacillin sodium and sulbactam sodium was initiated. On June 29^th^, the patient's symptoms alleviated, and he was discharged from the hospital.

## 3 DISCUSSION

In this study, two pediatric patients (with a total of three episodes) exhibited typical abnormal signals in the splenium of the corpus callosum and bilateral corona radiata on MRI, accompanied by reversible neurological dysfunction. Given their ages and disease courses, which were consistent with the characteristics of CLOCC, they were diagnosed with CLOCC. In this study, case 1 experienced onsets at ages 12 and 14, with varying clinical and radiological manifestations between the two episodes, both accompanied by left-sided limb weakness. This similarity in clinical manifestations across multiple episodes is also observed in previous reports [4], suggesting that patients may have a specific underlying susceptibility or cause. The recurrence of the rare disease CLOCC implies the involvement of certain genetic defects in its pathogenesis. This study involves a pair of siblings, and it remains uncertain whether CLOCC is associated with genetic factors. Previous studies have revealed associations between certain genetic defects and CLOCC. For instance, Gatto *et al.* found that mutations in cluster of differentiation 36 may be associated with CLOCC [3]. Kurahashi *et al.* suggested that variations in the myelin regulatory factor gene might be involved in the onset of familial CLOCC [5]. Ito *et al.* reported two brothers, both affected by CLOCC, who were subsequently diagnosed with X-linked Charcot-Marie-Tooth disease type 1, a genetic disorder [6]. Suzuki *et al.* described a pair of siblings with hereditary spherocytosis who concurrently developed CLOCC following infection with human parvovirus B19 [7].

In this study, the neurological symptoms of the two pediatric patients (with a total of three episodes) rapidly resolved after lasting for approximately one week. Similar to our study, Ito *et al.* reported a case of CLOCC with tetraplegia and dysarthria [6], and Tahara *et al.* reported a case of CLOCC with upper limb weakness [2]. In addition, previously reported neurological symptoms of CLOCC have also included disturbed consciousness, abnormal behavior, and delirium, among others [8, 9]. These highly heterogeneous clinical manifestations indicate the lack of specific symptoms in CLOCC. The CLOCC case reported by Shekari *et al.* [8] presented with transient visual loss, suggesting that involvement of the visual pathway might exist as a rare manifestation; however, no visual disturbances were observed in the cases of our study. Both case 1, before its first onset, and case 2, before its onset, had symptoms of rhinorrhea. During the first hospitalization, case 1 presented with dizziness and vomiting, and during the second hospitalization, cough was also present. Case 2 experienced fever and vomiting during the hospital stay. These symptoms are likely related to infection and intracranial pathology. According to a study by Chen *et al.*, the majority of pediatric patients with CLOCC (127/130, 97.7%) exhibited prodromal symptoms of infection, with respiratory infection symptoms being more common in children older than 3 years [10]. In this study, both patients recovered after treatment, indicating a favorable prognosis, which is consistent with the majority of previous studies. However, there have also been reported cases of pediatric CLOCC patients with residual neurological sequelae [11], thus aggressive treatment and monitoring remain necessary.

In previous cases of CLOCC in siblings, serum inflammatory markers have been reported to be either elevated [3] or within normal range [2]. In this study, the white blood cell count and CRP levels were not significantly elevated at admission for both patients (with a total of three episodes). Only in case 2, on the 8^th^ day after onset, the blood routine test showed a slight increase in white blood cell count and CRP, which seems to contradict the conclusion that inflammatory factors are significantly elevated in the blood of pediatric patients with CLOCC. Similar to our study, Tahara *et al.* also reported no significant abnormalities in the blood laboratory test results of the pediatric patients [2]. However, in the study by Aksu *et al.*, of 41 pediatric patients with sporadic callosal cytotoxic lesions, the median CRP level was 15.5 mg/L (normal level <5 mg/L) in 22 patients with infection-related conditions [12]. In the study by Chen *et al.*, most pediatric patients with CLOCC had elevated white blood cell counts and/or high-sensitivity CRP levels [11]. In this study, multiple CSF tests showed no significant abnormalities in the two patients. In previous case reports of CLOCC in siblings, CSF laboratory test results showed no significant abnormalities [1, 2, 7]. In two studies of sporadic CLOCC (including a total of 29 lumbar punctures), the CSF tests of the majority of patients showed no significant positive findings [11, 12]. Previous studies have reported an association between hyponatremia and CLOCC, with hyponatremia potentially leading to cerebral edema. In the study by Gatto *et al.*, one of the twin sisters with CLOCC had mild hyponatremia (132 mmol/L) [3]. In the study by Chen *et al.*, 30 out of 130 Chinese pediatric patients with CLOCC developed hyponatremia (ranging from 128-134 mmol/L) [10]. However, neither of the two patients in this group (with a total of three episodes) exhibited hyponatremia. No evidence of hypoglycemia was found in the cases studied. However, existing literature has indicated that hypoglycemia can induce cytotoxic edema, leading to similar imaging manifestations [13].

The imaging findings of the two patients (with a total of three episodes) in this study showed inconsistent locations and extents of the lesions, primarily involving the splenium of the corpus callosum and the deep white matter at the junction of the bilateral fronto-parietal lobes. The imaging manifestations were similar to those reported in case 2 by Imamura *et al.* [1] and in a pair of twin brothers reported by Tahara *et al.* [2]. In previous cases of CLOCC in siblings, significant variations have been observed in the extent of deep white matter involvement [1-3]. CLOCC is a clinical-radiological syndrome with an unknown specific pathogenesis; however, it is known to be a secondary lesion triggered by various diseases, including infections, tumors, drug reactions, and metabolic disorders [10, 14]. A pathological examination of a case reported by Hayashi *et al.* suggested that cytotoxic edema may be the central pathological mechanism of CLOCC [15]. The study by Chen *et al.* showed that infectious causes are the most common in pediatric patients with CLOCC, with rotavirus and Mycoplasma pneumoniae infections being the predominant ones [10]. In this study, although no definitive pathogens were detected, both patients (with a total of three episodes) had flu-like symptoms or prodromal symptoms, leading to the primary consideration of viral infection. Although it is speculated that genetic background may influence the susceptibility to CLOCC, definitive conclusions require in-depth genetic analysis. Currently, there is no specific treatment for CLOCC, and therapy primarily focuses on treating the underlying causes (such as anti-infective treatment) and providing symptomatic treatment (such as using mannitol to reduce intracranial pressure) [10, 16]. Given that CLOCC may be associated with inflammatory factors, steroid therapy has often been employed in previous studies [10, 17, 18]. The treatment of the cases in this study is not particularly different from that in previous studies. Although identifying the cause or underlying disease in pediatric patients with CLOCC can sometimes be challenging, this does not necessarily affect the treatment plan, as CLOCC is often self-limiting, and treatment primarily focuses on symptom relief.

## 4 STUDY LIMITATIONS

Limitations of this study include its retrospective nature, the lack of follow-up cranial MRI examinations after the treatment of the second episode in case 1 and after the treatment in case 2, the inability to definitively identify the pathogen, and the absence of further genetic testing.

## CONCLUSION

In conclusion, we report two cases of CLOCC occurring in two brothers successively, with one experiencing two episodes, suggesting a potential genetic association in the occurrence of CLOCC in children.

## Data Availability

The datasets generated during and/or analysed during the current study are available from the corresponding author [J.H] on reasonable request.

## LIST OF ABBREVIATIONS

CLOCC: Cytotoxic Lesions of the Corpus Callosum
MRI: Magnetic resonance imaging
RESLES: Reversible splenial lesion syndrome
DWI: Diffusion weighted imaging
FLAIR: Fluid-attenuated inversion recovery
EEG: Electroencephalogram
CSF: Cerebrospinal fluid

## STANDARDS OF REPORTING

CARE guidelines were followed.
